# Medical gas: dual role in ocular treatment and diagnostic applications

**DOI:** 10.4103/mgr.MEDGASRES-D-25-00174

**Published:** 2026-08-03

**Authors:** Nur Syafiqah Mat Zain, Lai Kuan Lee

**Affiliations:** Food Technology Program, School of Industrial Technology, Universiti Sains Malaysia, Pulau Pinang, Malaysia

##  

Medical gas therapy includes a variety of modalities of various gases. Gas treatment has been extensively studied for therapeutic uses. Recent research reveals that adjusting the supply of carbon dioxide, oxygen, and other medicinal gases can improve imaging modalities, assess retinal function, and aid in early ophthalmologic illness detection. This perspective explores the dual roles of the medical gas approach in both treatments and diagnostic applications in ophthalmology, emphasizing its possibility for improved clinical results.

**Medical gas therapy for the treatment of ocular diseases: *Hyperbaric oxygen therapy (HBOT):*** HBOT functions to deliver persistent (4 hours) oxygen at pressures > 1 atmosphere absolute to enhance tissue oxygenation. Extensive research revealed that HBOT improved central retinal artery occlusion, obstructive vasculopathies, vascular-induced cystoid macular edema, scleral necrosis, resistant corneal edema, anterior segment ischemia, sickle cell vitreoretinopathies, and treatment-resistant optic neuropathies.^1^ In ischemic eye disorders, intraocular HBOT elevates oxygen saturation within the vitreous body and protects retinal neurons from apoptosis.^2^ Mechanistically, HBOT increases the oxygen diffusion gradient from the choroidal circulation to the outer retinal layers, potentially supporting improved retinal oxygenation.

Nevertheless, the use of HBOT may increase reactive oxygen species and be linked to the development of myopia and cataracts. Furthermore, relative ischemia following HBOT sessions has been linked to increased release of vascular endothelial growth factor and enhanced vascular permeability in different retinal disorders.^1^ Additionally, using an oronasal mask instead of a hood can be a safer delivery method to mitigate specific risks like damage to the trabecular meshwork in glaucoma patients.^2^ An alternative to HBOT is normobaric oxygen therapy, which provides sufficient retinal oxygenation in less severe cases without the need for high-pressure utilization. While HBOT seems potential, clinical adoption is hampered by contradictory evidence, limited sample sizes, and a lack of long-term data. Future studies should focus on well-designed, carefully strategized randomized controlled trials to unravel its significance in ophthalmology.

***Oxygen-ozone (O_2_-O_3_) therapy:*** Promising outcomes following the application of O_2_-O_3_ therapy have been observed in Sjögren’s syndrome, age-related macular degeneration, and diabetic neuropathy. For Sjögren’s syndrome, a case report from Italy demonstrated a successful administration of the therapy for the improvement of ocular dryness, fatigue, and pain. The successful therapy worked by promoting mitochondrial biogenesis, in addition to the restoration of a balanced immune response in Th17/Treg homeostasis. A special characteristic of the alternative route was administering the O_2_-O_3_ gas mixture directly into the rectum.^3^ In 12 age-related macular degeneration patients who were provided with autohemoinfusion, 150 mL of the patient’s blood was combined with 150 mL of O_2_-O_3_. The observed therapeutic advantages included an improved cellular oxygenation and metabolism, which potentially increasing the function of retinal cells responsible for visual acuity.^4^ However, concerns remain regarding the lack of standardized treatment protocols, technical demands for safe delivery, limited long-term follow-up data, and the absence of large-scale confirmatory clinical trials. Overall, O_2_-O_3_ therapy represents a novel adjunctive medical gas therapy with the unique functions to elevate oxygen utilization, reduce oxidative burden, and modulate immune responses.

***Ozone (O_3_) therapy:*** In retinitis pigmentosa patients, rectal administration of 10 mg O_3_ gas via a catheter has shown short-term improvements in visual field area compared with oxygen alone, particularly in early-stage disease.^5^ The effect is thought to be mediated through enhanced tissue oxygenation, modulation of oxidative stress, and stimulation of endogenous antioxidant defenses. According to Tratsk, improvements have been observed in a 73-year-old woman with open-angle glaucoma, diabetic retinopathy, and other serious retinal conditions after O_3_ therapy.^6^ A good response and improvement in visual acuity, and the considerable results implied that O_3_ therapy can restore eye function and improve the quality of life. The introduction of O_3_ is believed to provide disinfecting and antioxidant properties, increases blood circulation, eliminates toxins, and improves organ function.

***Hydrogen (H_2_) and hydrogen sulfide (H_2_S) therapies:*** In a prospective randomized trial, H_2_ gas was dissolved into the standard ocular irrigation solution (Ope Guard neo) by placing the solution bag in a vacuum chamber (100% H_2_) gas for 24 hours during phacoemulsification. Results showed that H_2_ therapy reduced the corneal endothelial damage by selectively scavenging hydroxyl radicals (·OH), and reduced endothelial cell density loss. Such initiative was viable to reduce corneal endothelial damage during phacoemulsification, the most reported complication due to cataract surgery.^7^ However, the challenges of maintaining sufficient dissolved H_2_ concentrations in H_2_-rich water, H_2_ loss and imprecise dosing following oral administration remained the typical drawbacks. Additionally, the explosive and low solubility characteristics of the H_2_ gas require safety precautions before wider clinical adoption.

H_2_S is an emerging therapeutic gas for ocular diseases due to its antioxidant and anti-inflammatory properties. In age-related macular degeneration, H_2_S-based preconditioning mitigates chronic injury by reducing oxidative stress and suppressing inflammatory pathways, thereby preserving retinal integrity.^8^ H_2_S and its donors also regulate oxidative stress in retinal degenerative diseases, helping to alleviate retinopathy by counteracting its damaging effects. In glaucoma, H_2_S showed potential in lowering intraocular pressure and protecting retinal ganglion cells by scavenging reactive oxygen species, increasing glutathione, reducing excitotoxicity, relaxing ciliary muscles, and enhancing trabecular outflow. Despite these therapeutic benefits, clinical application remains limited due to challenges in stability, delivery, and safety.^9^

***Inert gases:*** Inert gases such as sulfur hexafluoride (SF_6_), perfluoroethane (C_2_F_6_), and octafluoropropane (C_3_F_8_) have been increasingly utilized to promote chorioretinal adhesion. In vitrectomy procedures, intraocular gas tamponade acts as a bubble that helps keep the retinal surface dry as it heals properly. The bubble is delivered into the eye to help seal the macular hole, which permits the retinal pigment epithelium to absorb any leftover subretinal fluid and promote retinal reattachment. Nevertheless, the supply of intraocular gases can lead to increased intraocular pressure, cataract development, gas migration, and temporary visual disturbances, probably due to the difference in refractive indices between gas and lens.^10^

***Nitric oxide (NO):*** NO acts as a beneficial therapeutic agent by regulating intraocular pressure. A previous study revealed a significant 66% increase in NO production when pressure rose from 10 mmHg to 25 mmHg.^11^ The NO increase is partially reversible with L-NAME, a nitric oxide synthase inhibitor, and is associated with increased inducible nitric oxide synthase transcript levels.^11^ However, inducible nitric oxide synthase presents a significant concern, as its well-established role in inflammatory pathways raises the possibility of unintended pro-inflammatory effects within the eye. However, the therapeutic use of NO lay the safety concern, as excessive levels of NO lead to nitrosative stress, a medical condition contributing to dry eye syndrome and keratitis.

**Gas-based ocular diagnostic applications: *O_2_-CO_2_ modulation:*** Xu et al.^12^ demonstrated that breathing 80% oxygen caused a significant reduction in perfused vessel density in the macular capillaries (~15%) compared to the peripapillary region (~9.5%). Such an application highlighted that hyperoxic response could serve as a safe and reliable tool for early identification of compromised retinal autoregulation. Additionally, O_2_ remarkably lowered blood flow velocity and retinal vascular diameter, while CO_2_ increased the blood flow velocity without changing the vessel size. Such findings aid in the early detection and management of a variety of retinal vascular disorders.

***CO_2_-based diagnostic:*** The application of CO_2_ stimulation to study tear flow dynamics has produced interesting results. In 2004, De Paiva and Pflugfelder evaluated corneal sensation on mechanical (air jet) and chemical using a modified Belmonte gas esthesiometer in normal subjects, dry eye patients, and post-laser-assisted in situ keratomileusis patient with or without dry eye. Mechanical stimulation involved a 2-second pulse of warm air (50°C) to the central cornea, starting at 10 mL/min, and increased by 20 mL/min until the sensation was detected. The flow was then reduced in smaller steps to determine the lowest detectable threshold. Whilst chemical stimulation was performed by increasing CO_2_ concentration in the air jet, using an airflow of 10 mL/min below the mechanical threshold. The Belmonte modified noncontact esthesiometer is an advanced tool that assesses corneal sensitivity by stimulating different types of sensory receptors using controlled air jets. The tool was effective at detecting subtle changes in sensitivity associated with corneal surface damage, making it a valuable instrument and useful efficacy parameter in clinical trials for patients with dry eye disease and keratitis sicca.^13^ Both the O_2_-CO_2_ and CO_2_-based systems are not interchangeable as they test distinct physiological pathways. An O_2_-based system evaluates autoregulatory vasoconstriction, while the CO_2_-based system measures vasodilation and neurovascular coupling. A combined system provides a more comprehensive evaluation and offering a complete assessment of vascular health.

**Conclusion:** In the realm of ophthalmology, medical gas therapy has shown a profound effect in both therapeutic and diagnostic applications (**Figure 1**). O_2_, CO_2_, O_3_, H_2_, H_2_S, NO, and inert gases demonstrated distinct capabilities in treating various eye diseases. However, the full potential of gas-based intervention remains underexplored, particularly in the field of diagnostics. The diagnostic use of medical gases in ophthalmology is a growing area that offers important insights into ocular physiology and disease causes. Integrating gas-responsive imaging techniques and non-invasive gas stimulation tests may pave the way for earlier disease detection and improved clinical outcomes. While the current literature has revealed promising effects of gas-based interventions for eye disease, larger research with optimized techniques is required to ascertain the efficacy.

**Figure 1:**
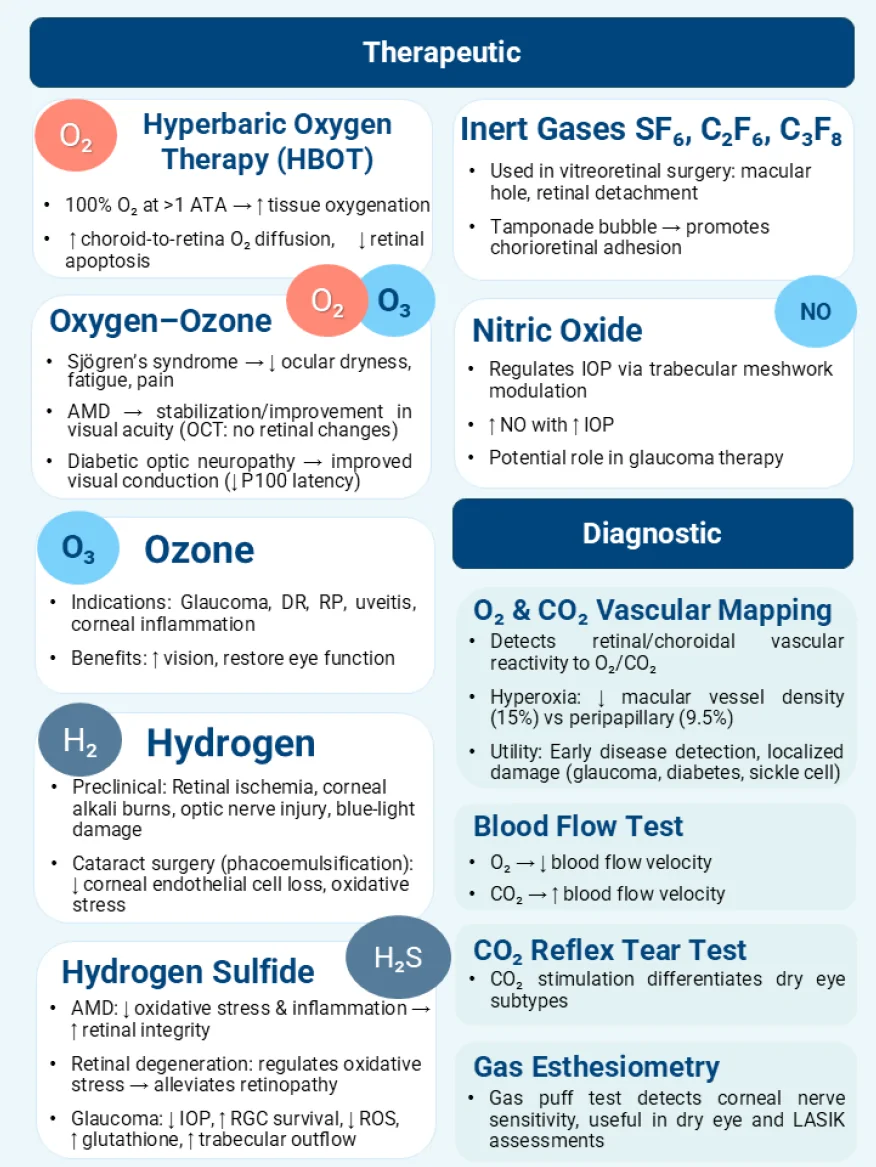
Roles of medical gases in ocular treatment and diagnostic applications. Therapeutic roles consist of hyperbaric oxygen therapy, oxygen–ozone therapy, ozone therapy, hydrogen therapy, inert gases, and NO. For diagnostic application, vascular mapping, blood flow testing, CO_2_ reflex tear testing, and gas esthesiometry utilized oxygen and carbon dioxide have been applied. Created with Microsoft PowerPoint 2013 MSO. AMD: Age-related macular degeneration; ATA: atmosphere absolute; CO_2_: carbon dioxide; DR: diabetic retinopathy; HBOT: hyperbaric oxygen therapy; IOP: intraocular pressure; LASIK: laser-assisted *in situ* keratomileusis; NO: nitric oxide; OCT: optical coherence tomography; RGC: retinal ganglion cell; ROS: reactive oxygen species; RP: retinitis pigmentosa.

*We acknowledge the research support services provided by Universiti Sains Malaysia*.

*The authors declare that no generative AI was used in the preparation of this manuscript.*
